# Geographic Disparities in Access to Assisted Reproductive Technology Centers in China: Spatial-Statistical Study

**DOI:** 10.2196/55418

**Published:** 2024-06-12

**Authors:** Qingqing Zhou, Huatang Zeng, Liqun Wu, Kaichuan Diao, Rongxin He, Bin Zhu

**Affiliations:** 1 School of Public Health and Emergency Management Southern University of Science and Technology Shenzhen China; 2 Shenzhen Health Development Research and Data Management Center Shenzhen China; 3 Vanke School of Public Health Tsinghua University Beijing China

**Keywords:** assisted reproductive technology, spatial accessibility, travel time, travel cost, China

## Abstract

A study on infertility in China found that while 543 health care institutions are approved for assisted reproductive technology (ART), only 10.1% offer all ART services, with a significant skew toward the eastern regions, highlighting the accessibility challenges faced by rural and remote populations; this study recommends government measures including travel subsidies and education initiatives to improve ART access for economically disadvantaged individuals.

## Introduction

Infertility is a growing, serious public health concern [1]. In 2023, the World Health Organization reported that infertility affects about 17.5% of the adult population globally [2]. Infertility is also prominent in China, affecting nearly 50 million people [3]. However, the travel time and costs are often a significant burden faced by patients with infertility [4,5]. Here we used a web path planning engine [6] to explore the spatial-economic disparities in access to assistive reproductive technology (ART) centers in mainland China.

## Methods

### Study Design

The National Health Commission of the People’s Republic of China supplied a list of ART centers, while population data were sourced from the 2020 Worldpop data set [7]. Residential point locations were gathered from the Gaode Maps open platform. After retaining one residential point within a 1-km radius, 57,469 residential points were acquired. We opted for a contemporary approach by leveraging real-time traffic data from web map navigation services—a departure from traditional methods. Using the Path Planning 2.0 algorithm, we determined optimal paths among residential points and the nearest ART center, predicting detailed travel times and corresponding costs [8,9]. We averaged the travel times and costs of residential points within the county, thus representing its overall level. Subsequently, isochronous maps depicting 1-hour and 2-hour travel times for ART services were generated.

### Ethical Considerations

This study used deidentified publicly available data sets, all aggregated at the county level. Per Article 3 of the Southern University of Science and Technology’s institutional review board guidelines, we did not require peer review as this is a secondary analysis using publicly accessible locations of ART centers from the National Health Commission’s website and publicly accessible locations of residential points. These data sets contain no personally identifiable information and pose no risk of ethical violation.

## Results

**Figure 1:**
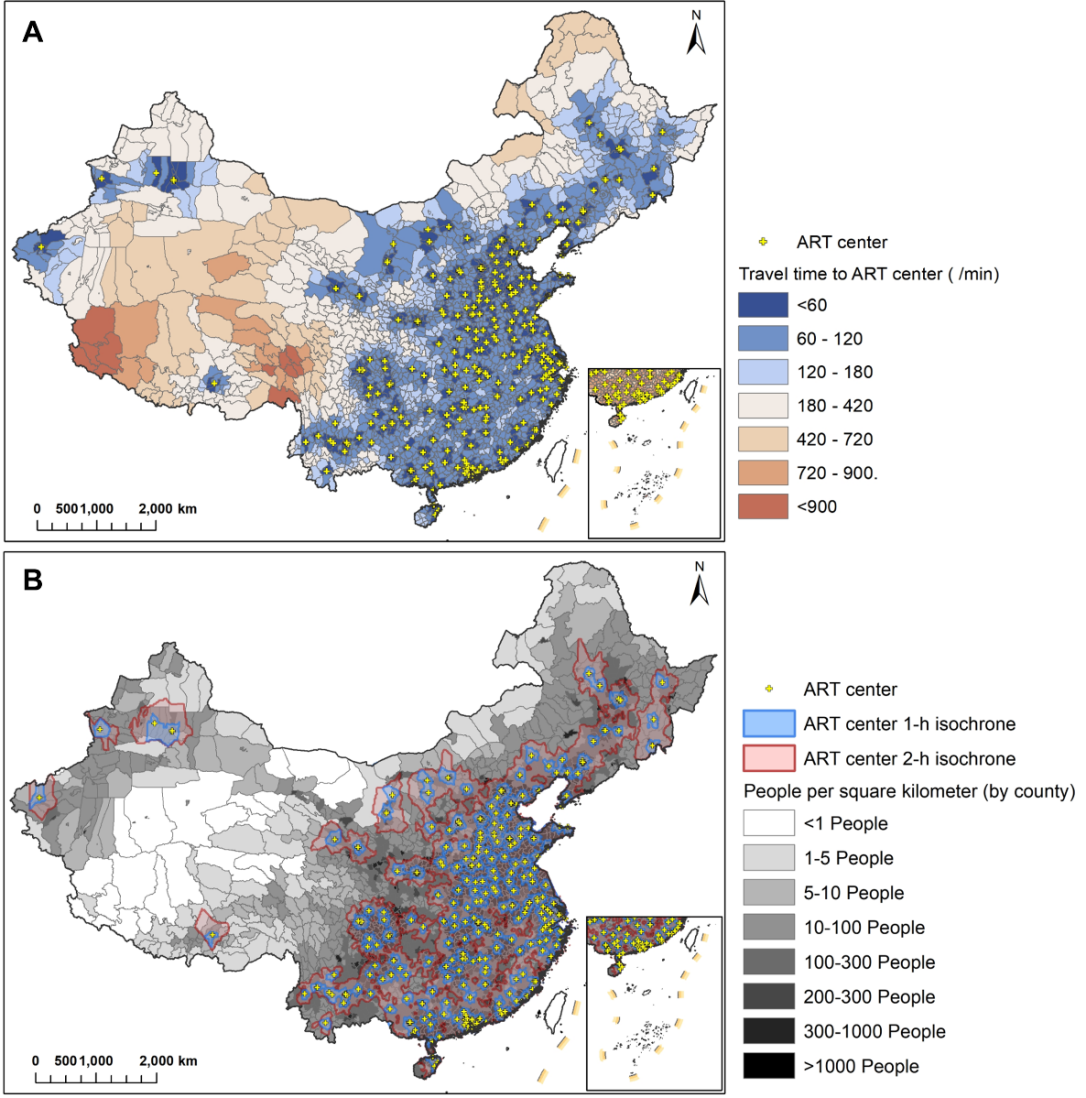
Travel time to assisted reproductive technology (ART) centers in China. (A) A map of travel times to ART centers at the county level. (B) The population density of China's counties covering 1-hour and 2-hour travel time isochrones. Counties with higher population densities are shaded in dark gray. Areas shaded in red are located within the 1-hour and 2-hour travel time isochrones of the ART centers.

As of June 2022, in total, 543 health care institutions in Mainland China have received approved to conduct ART. However, only 55 (10.1%) institutions offer all 5 types of ART services. The distribution of ART centers in China predominantly favors the eastern plains and coastal regions (Figure 1A). Among 7 geographical subregions of China, East China boasts the highest number of ART centers (n=162, 29.8%). Specifically, Guangdong province leads with the greatest number of ART centers (n=56, 10.3%), with 83.9% (n=104) of the province’s counties accessible to ART centers within 1.5 hours and an average taxi cost of merely 66.7 CNY (US $9.21) [10]. In contrast, the Northwest region has the lowest number of ART centers (n=27, 5.0%). Tibet, in particular, has only 1 ART center, and a mere 12.2% (n=9) of Tibetan counties have access to it within 1.5 hours, with taxi costs soaring as high as 1485.2 CNY (US $205.08).

Travel time and costs to ART centers by province are presented in Table 1. Detailed information on travel time and costs is visualized in Multimedia Appendix 1. In China, a substantial portion of the population faces challenges in accessing ART facilities within a short time frame. Specifically, 76.8% (n=180,330,033) of the total population and 63.5% (n=36,390) of residential points are not reachable to an ART center within an hour.

**Table 1:** Travel time and travel costs to assistive reproductive technology (ART) centers by province and proportion of population access to ART services at different time thresholds.

Province		ART centers, n	Travel time (minutes)	Cost (CNY^a^)	Population (%)		
					≤30 minutes	≤60 minutes	≤120 minutes
**North**							
	Beijing	18	20.8	52.2	80.7	97.7	100
	Tianjin	12	22.5	38.5	78.7	93.9	99.9
	Hebei	31	43.0	93.1	44.3	75.5	94.1
	Shanxi	12	45.0	112.1	49.2	65.1	96.7
	Inner Mongolia	8	89.3	237.3	44.0	55.9	70.5
**Northeast**							
	Heilongjiang	11	63.0	151.3	52.9	64.0	79.3
	Jilin	9	72.4	153.3	40.0	52.3	77.3
	Liaoning	19	47.5	90.3	44.1	69.4	94.9
**East**							
	Shanghai	20	17.0	49.9	86.9	99.5	100
	Jiangsu	33	38.0	71.6	42.9	83.8	100
	Zhejiang	27	40.0	90.1	35.5	81.6	99.8
	Anhui	16	44.8	101.8	46.2	63.2	97.8
	Jiangxi	18	50.4	126.9	41.7	59.0	96.1
	Shandong	32	38.1	90.0	44.1	79.5	100
	Fujian	16	36.2	108.2	52.2	83.7	97.6
**Central**							
	Henan	33	38.8	66.6	45.5	97.3	99.5
	Hunan	24	52.0	121.0	39.2	59.8	95.6
	Hubei	32	39.5	68.2	49.5	72.1	98.6
	Southern						
	Guangdong	56	27.7	66.7	67.6	91.2	99.5
	Guangxi	21	42.3	102.0	53.0	65.1	98.0
	Hainan	10	58.3	246.1	50.9	60.0	75.1
**Southwest**							
	Chongqing	12	44.9	153.0	44.9	68.4	95.6
	Guizhou	13	46.2	99.9	49.1	66.1	94.4
	Sichuan	14	50.5	140.2	44.6	70.5	91.7
	Yunnan	18	69.8	199.2	44.4	59.1	77.3
	Tibet	1	267.9	1485.2	47.3	53.0	58.2
**Northwest**							
	Ningxia	2	63.2	119.3	41.9	61.1	81.6
	Qinghai	2	82.8	190.8	60.2	77.7	85.9
	Gansu	4	131.9	157.1	29.5	32.6	49.3
	Shaanxi	10	64.6	179.2	38.5	58.4	79.3
	Xinjiang	9	108.4	275.5	56.3	61.5	67.1

^a^1CNY=US $0.14. Travel cost was determined by the local taxi fare, which varies depending on the location. The first price covers a specified distance, often 2.5-3 km, and every additional kilometer is then calculated at a certain price.

## Discussion

The distribution of ART centers in China exhibits significant disparities. A higher concentration of ART centers is observed in urban and eastern regions, while individuals in northwestern and rural areas encounter prolonged travel times and elevated transportation costs when seeking ART treatment. The map serves the dual purpose of estimating the likelihood of individuals seeking ART treatment when needed and providing an evidence-based foundation for efficient allocation of limited ART resources to underserved populations (present and future). The government is suggested to implement a series of measures, including counterpart aid and effective initiatives to educate and recruit ART doctors in disadvantaged units. Residents in rural and remote areas contend with extended travel times and substantial travel costs when accessing ART services, which should be covered fully or partially through travel subsidies or paid leaves. The introduction of telehealth services is a viable solution to surmount these barriers for patients residing in remote areas, effectively reducing in-person office visits. Notably, ensuring privacy is paramount when helping patients seeking ART treatments. This study bears limitations. While we used the best available data, the residential point data sets remain susceptible to omission errors, which confines the algorithm’s usage to only 1 mode of transportation—a limitation not potentially aligning with real-world scenarios. Additionally, individuals may not necessarily receive ART treatment at the nearest facility or may opt for alternative transportation means.

## Appendix

Multimedia Appendix 1 Travel time and cost analyses.

## Abbreviations

ART: assisted reproductive technology
