# Supplementary material for: Geographic Disparities in Access to Assisted Reproductive Technology Centers in China: Spatial-Statistical Study
Source: JMIR Public Health Surveill. 2024 Jun 12;10:e55418. doi: 10.2196/55418 (PMC11208828; doi:10.2196/55418)
Supplement: Multimedia Appendix 1 [file publichealth_v10i1e55418_app1.docx]

**Supplementary Appendix**

**Technical Note S1. Computation of County's time and cost**

Utilizing the Gaode Maps Open Platform API (https://lbs.amap.com/), we batch specified the latitude/longitude coordinates of both the origin (residential points) and destination (ART centers), and then calculated the optimal path between them. After obtaining the time and cost for each origin and destination point, the time and cost for the entire county are determined using Equation 1, 2.

| ${time}_{county}=\frac{\sum_{i} (t_{i}\times p_{i})}{\sum_{i} p_{i}}$ | (1) |
| --- | --- |
| ${cost}_{county}=\frac{\sum_{i} (c_{i}\times p_{i})}{\sum_{i} p_{i}}$ | (2) |

where ${time}_{county}$ represents county-level time; ${cost}_{county}$ represents county-level cost; $t_{i}$ and $c_{i}$ represent the time and cost of the $i$ resident, and $p_{i}$ represents the population density of the $i$ settlement.

**eFigure 1.** Travel Time to Healthcare Institutions Providing Five Different Assisted Reproductive Technology.


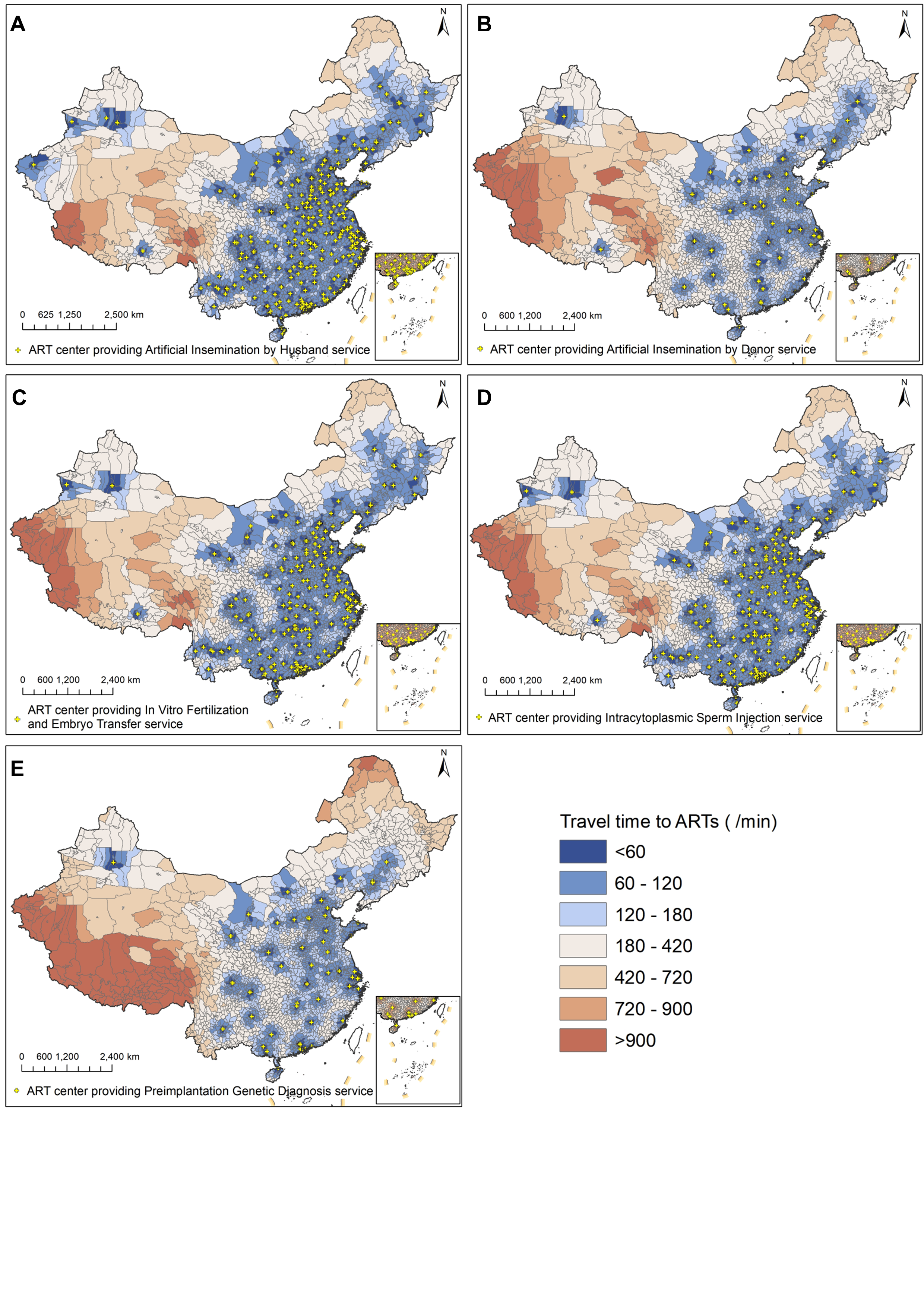


**eFigure 2.** Travel Cost to Healthcare Institutions Providing Five Different Assisted Reproductive Technology.


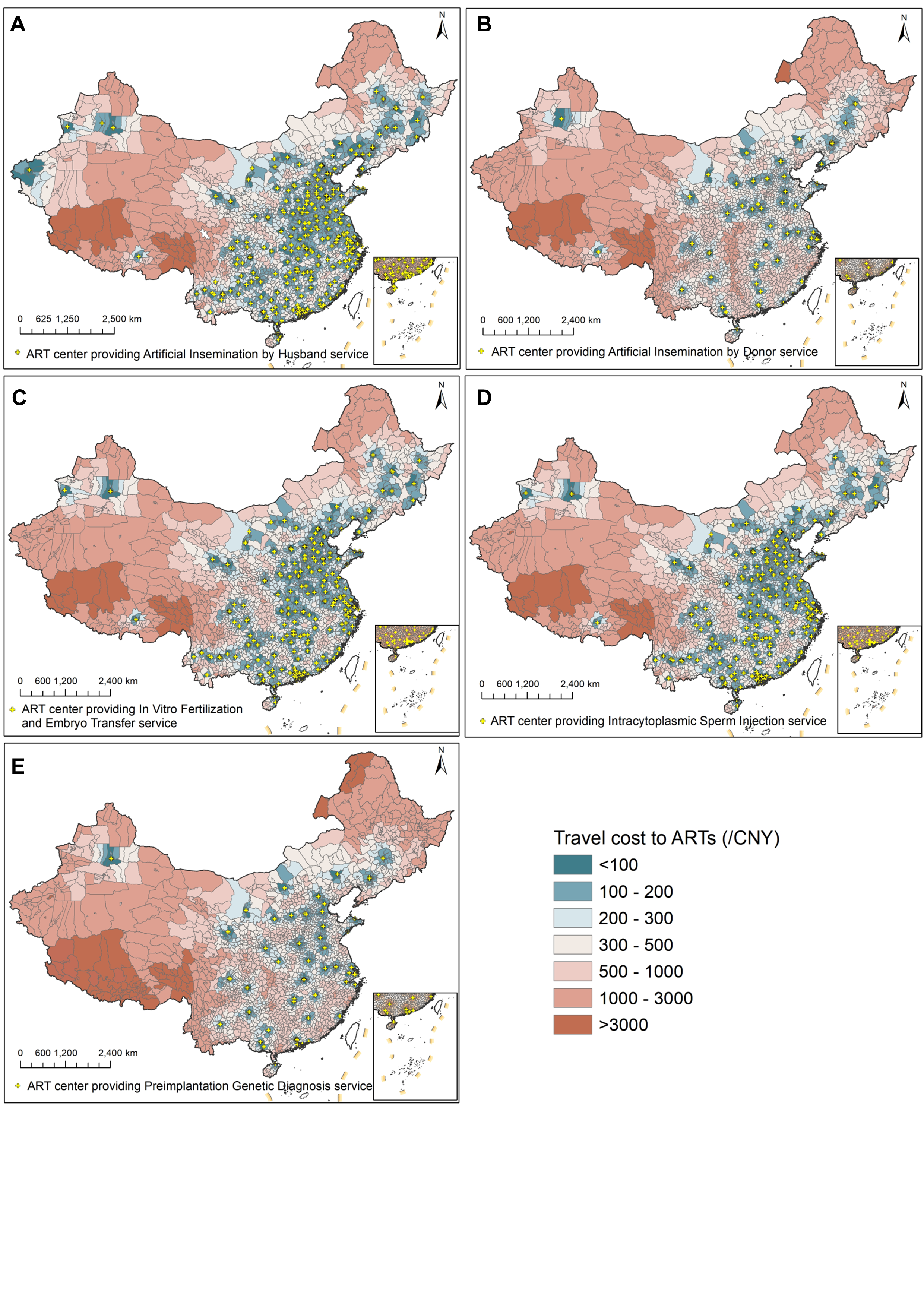


**eFigure 3**. The Percentage of the National Population that Could Assess to the Five Types of ART Services at Different Time Thresholds.


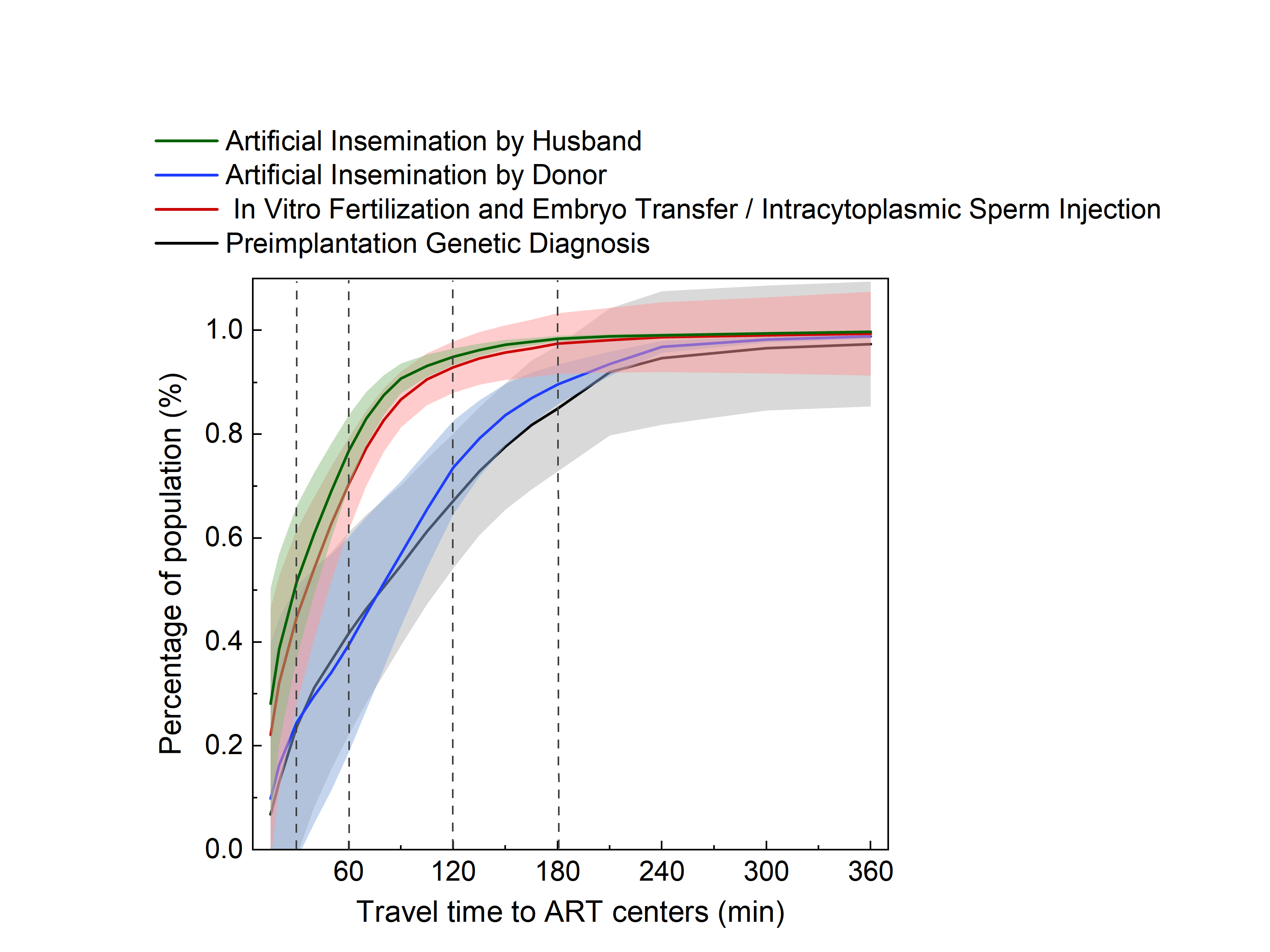


**eTable.** Travel Time and Travel Costs to the ART Centers with 5 Different Technologies by Province.

| **Province** | **AIH** | | | **AID** | | | **IVF-ET** | | | **ICSI** | | | **PGD** | | |
| --- | --- | --- | --- | --- | --- | --- | --- | --- | --- | --- | --- | --- | --- | --- | --- |
|  | **Number** | **Time (min)** | **Cost (CNY)** | **Number** | **Time (min)** | **Cost (CNY)** | **Number** | **Time (min)** | **Cost (CNY)** | **Number** | **Time (min)** | **Cost (CNY)** | **Number** | **Time (min)** | **Cost**  **(CNY)** |
| North |  |  |  |  |  |  |  |  |  |  |  |  |  |  |  |
| Beijing | 18 | 20.8 | 52.2 | 4 | 23.5 | 62.1 | 16 | 26.4 | 49.1 | 16 | 26.4 | 49.1 | 3 | 33.7 | 62.4 |
| Tianjin | 12 | 22.5 | 38.5 | 0 | 93.2 | 289.6 | 8 | 28.4 | 48.8 | 8 | 28.4 | 48.8 | 1 | 40.3 | 55.9 |
| Hebei | 31 | 43.0 | 93.1 | 2 | 94.7 | 304.4 | 24 | 50.7 | 83.8 | 24 | 50.7 | 83.8 | 2 | 99.9 | 251.3 |
| Shanxi | 12 | 45.0 | 112.1 | 3 | 89.9 | 225.0 | 11 | 53.2 | 139.9 | 11 | 53.2 | 139.9 | 1 | 118.8 | 383.4 |
| Inner mongolia | 8 | 89.3 | 237.3 | 2 | 140.5 | 456.8 | 6 | 96.0 | 301.4 | 6 | 96.0 | 301.4 | 2 | 156.8 | 490.4 |
| Northeast |  |  |  |  |  |  |  |  |  |  |  |  |  |  |  |
| Heilongjiang | 11 | 63.0 | 151.3 | 2 | 141.1 | 425.8 | 8 | 68.4 | 178.3 | 8 | 68.4 | 178.3 | 0 | 290.1 | 978.1 |
| Jilin | 9 | 72.4 | 153.3 | 1 | 106.7 | 281.3 | 6 | 69.0 | 179.2 | 6 | 69.0 | 179.2 | 1 | 119.7 | 284.2 |
| Liaoning | 19 | 47.5 | 90.3 | 7 | 82.8 | 227.0 | 12 | 60.6 | 181.5 | 12 | 60.6 | 181.5 | 3 | 132.3 | 459.4 |
| East |  |  |  |  |  |  |  |  |  |  |  |  |  |  |  |
| Shanghai | 20 | 17.0 | 49.9 | 9 | 24.1 | 48.1 | 20 | 18.4 | 55.4 | 20 | 18.4 | 55.4 | 8 | 21.7 | 49.0 |
| Jiangsu | 33 | 38.0 | 71.6 | 6 | 74.5 | 207.4 | 26 | 35.1 | 79.0 | 26 | 35.1 | 79.0 | 7 | 72.1 | 197.2 |
| Zhejiang | 27 | 40.0 | 90.1 | 2 | 123.9 | 537.2 | 19 | 46.0 | 112.0 | 19 | 46.0 | 112.0 | 3 | 96.1 | 488.1 |
| Anhui | 16 | 44.8 | 101.8 | 3 | 85.1 | 266.8 | 9 | 58.6 | 120.2 | 9 | 58.6 | 120.2 | 3 | 86.2 | 205.6 |
| Jiangxi | 18 | 50.4 | 126.9 | 2 | 108.2 | 379.0 | 15 | 50.4 | 166.6 | 15 | 50.4 | 166.6 | 2 | 97.7 | 302.7 |
| Shandong | 32 | 38.1 | 90.0 | 7 | 86.3 | 193.4 | 28 | 43.5 | 111.0 | 28 | 43.5 | 111.0 | 4 | 83.2 | 227.0 |
| Fujian | 16 | 36.2 | 108.2 | 4 | 63.2 | 240.7 | 12 | 47.1 | 112.7 | 12 | 47.1 | 112.7 | 2 | 146.2 | 519.7 |
| Central |  |  |  |  |  |  |  |  |  |  |  |  |  |  |  |
| Henan | 33 | 38.8 | 66.6 | 6 | 80.9 | 212.7 | 18 | 56.8 | 96.8 | 18 | 56.8 | 96.8 | 3 | 104.0 | 226.4 |
| Hunan | 24 | 52.0 | 121.0 | 4 | 104.2 | 371.4 | 21 | 51.0 | 132.6 | 21 | 51.0 | 132.6 | 3 | 132.8 | 406.9 |
| Hubei | 32 | 39.5 | 68.2 | 5 | 94.3 | 347.3 | 23 | 44.8 | 92.6 | 23 | 44.8 | 92.6 | 7 | 95.6 | 232.0 |
| Southern |  |  |  |  |  |  |  |  |  |  |  |  |  |  |  |
| Guangdong | 56 | 27.7 | 66.7 | 5 | 75.3 | 274.6 | 53 | 29.2 | 75.7 | 53 | 29.2 | 75.7 | 10 | 67.5 | 289.3 |
| Guangxi | 21 | 42.3 | 102.0 | 2 | 129.2 | 543.2 | 20 | 46.4 | 118.8 | 20 | 46.4 | 118.8 | 4 | 102.0 | 315.0 |
| Hainan | 10 | 58.3 | 246.1 | 1 | 78.2 | 357.0 | 7 | 65.8 | 229.6 | 7 | 65.8 | 229.6 | 1 | 95.9 | 319.8 |
| Southwest |  |  |  |  |  |  |  |  |  |  |  |  |  |  |  |
| Chongqing | 12 | 44.9 | 153.0 | 3 | 64.5 | 265.6 | 7 | 67.1 | 208.6 | 7 | 67.1 | 208.6 | 3 | 74.8 | 233.3 |
| Guizhou | 13 | 46.2 | 99.9 | 1 | 99.4 | 290.9 | 8 | 70.3 | 143.3 | 8 | 70.3 | 143.3 | 2 | 111.3 | 271.7 |
| Sichuan | 14 | 50.5 | 140.2 | 3 | 95.4 | 263.2 | 10 | 63.8 | 200.3 | 10 | 63.8 | 200.3 | 3 | 93.2 | 317.1 |
| Yunnan | 18 | 69.8 | 199.2 | 2 | 161.1 | 582.7 | 16 | 84.7 | 267.4 | 16 | 84.7 | 267.4 | 1 | 167.7 | 621.1 |
| Tibet | 1 | 267.9 | 1485.2 | 1 | 271.3 | 1575.9 | 1 | 277.6 | 1575.9 | 1 | 277.6 | 1575.9 | 0 | 1791.1 | 5624.1 |
| Northwest |  |  |  |  |  |  |  |  |  |  |  |  |  |  |  |
| Ningxia | 2 | 63.2 | 119.3 | 1 | 65.5 | 135.5 | 2 | 63.1 | 166.6 | 2 | 63.1 | 166.6 | 1 | 73.1 | 130.4 |
| Qinghai | 2 | 82.8 | 190.8 | 0 | 256.2 | 675.3 | 2 | 110.5 | 268.6 | 2 | 110.5 | 268.6 | 0 | 262.6 | 674.4 |
| Gansu | 4 | 131.9 | 157.1 | 3 | 146.0 | 381.0 | 4 | 146.3 | 285.7 | 4 | 146.3 | 285.7 | 2 | 157.1 | 322.0 |
| Shaanxi | 10 | 64.6 | 179.2 | 2 | 91.8 | 226.4 | 5 | 82.0 | 249.7 | 5 | 82.0 | 249.7 | 2 | 89.1 | 250.7 |
| Xinjiang | 9 | 108.4 | 275.5 | 1 | 352.5 | 742.2 | 5 | 280.2 | 601.7 | 5 | 280.2 | 601.7 | 2 | 328.1 | 734.8 |

**Notes:**

Abbreviations: AIH, Artificial Insemination by Husband; AID, Artificial Insemination by Donor; IVF-ET, In Vitro Fertilization and Embryo Transfer; ICSI, Intracytoplasmic Sperm Injection; PGD, Preimplantation Genetic Diagnosis.

a. Travel cost is determined by the local taxi fare, which varies depending on the location. The first price covers a distance, often 2.5–3 km; every additional kilometer is then calculated at a certain price.
